# The Modified Bushen Antai Recipe Upregulates Estrogen and Progesterone Receptors at the Maternal-Fetal Interface in Pregnant Rats with Mifepristone-Induced Pregnancy Loss

**DOI:** 10.1155/2019/8312020

**Published:** 2019-01-16

**Authors:** Li Sun, Zhengwei Yuan, Lingyan Jian, Qinghua Jiang, Siwen Zhang, Jichun Tan

**Affiliations:** ^1^Reproductive Medicine Center, Obstetrics and Gynecology Department, Shengjing Hospital Affiliated to China Medical University, Shenyang 110022, China; ^2^Key Laboratory of Reproductive Dysfunction Diseases and Fertility Remodelling of Liaoning Province, Shengjing Hospital Affiliated to China Medical University, Shenyang 110022, China; ^3^Key Laboratory of Health Ministry for Congenital Malformation, Shengjing Hospital Affiliated to China Medical University, Shenyang 110022, China; ^4^Department of Pharmacy, Shengjing Hospital of China Medical University, Shenyang 110022, China

## Abstract

**Background:**

The modified Bushen Antai recipe (BSAT) is a centuries-old traditional Chinese medicine that we use in our center as a therapy against pregnancy loss. Our study aimed to explore the potential benefit and mechanism of BSAT in pregnant rats with mifepristone-induced pregnancy loss.

**Materials and Methods:**

The signature compounds of the eight BSAT ingredients were analyzed by high-performance liquid chromatography (HPLC). The BSAT group (n = 8) was treated daily with 6.3 ml/kg BSAT from gestation day (D) 0.5 to 10.5 and once with 1.25 mg/kg mifepristone on D 10.5. Normal saline replaced BSAT in the model group (n = 8), and both BSAT and mifepristone in the control group (n = 8). Morphological and histological analyses were performed on D 13.5.

**Results:**

BSAT contains eight medicinal ingredients including* Cuscuta chinensis* and* Dipsacus asperoides*. The HPLC analysis detected the signature compounds of seven medicinal ingredients in the extract. Embryo resorption rate in the BSAT group was significantly lower than that in the model group, although the number of surviving embryos was similar between the two groups. Hematoxylin and eosin (HE) staining suggested that the maximum cross-sectional area of the placenta and the area ratio of the placental labyrinth in the BSAT group were higher than those in the model group. Immunohistochemical (IHC) staining indicated that the expression of ki67, estrogen receptor alpha (ER*α*), and progesterone receptor (PR) in the placental labyrinth of the BSAT group was higher than that of the model group. Furthermore, the protein levels of ER*α*, PR, phospho-Akt/Akt, and phospho-Erk1/2/Erk1/2 in the BSAT group were higher than those in the control group. The mRNA levels of ER*α* and PR in the BSAT group were higher than those in the control group.

**Conclusions:**

BSAT may induce estrogen and progesterone receptors by phosphorylation via the classic Akt and Erk1/2 signaling pathways in the maternal-fetal interface of pregnant rats, thereby reducing the pregnancy loss rate and improving the live birth rate.

## 1. Introduction

The pregnancy loss is defined as the termination of pregnancy before the 28th week of gestation or a fetal weight of less than 1000 g [1]. It affects approximately 20% of all recognized pregnancies [2]. Furthermore, 3%-16% of pregnancy loss will lead to an inevitable pregnancy loss due to an increase in vaginal bleeding or severe abdominal pain [3, 4].

Causes of pregnancy loss include embryonic, maternal, paternal, and environmental factors [5, 6]. Approximately 50%-60% of pregnancy loss is caused by fetal chromosomal abnormalities, the most common factor [7]. Maternal factors include systemic diseases, genital abnormalities, endocrine abnormalities, intense stress and bad habits, and immune dysfunction [8]. Studies have also shown that sperm chromosomal abnormalities can be associated with spontaneous pregnancy loss [9]. In addition, excessive exposure to radiation and chemicals such as arsenic, lead, formaldehyde, benzene, chloroprene, and ethylene oxide can cause pregnancy loss [5, 6, 10].

In current clinical practice, pregnancy loss is prevented and treated by empirical nonsurgical interventions [11]. Patients are usually advised to take bed rest and avoid sexual intercourse, but there is insufficient evidence showing that these measures are effective [12]. Progesterone therapy is widely used and proven to be effective but causes adverse reactions such as injection site infection and dizziness [13, 14]. Other therapeutics such as human chorionic gonadotropin (HCG) or cyclooxygenase inhibitors have also been used for treatment of pregnancy loss but have failed to reduce the incidence rate [15]. In recent years, traditional Chinese medicine (TCM), often based on herbal medicines, has been widely used as an effective intervention for pregnancy loss [16].

TCM differs from Western medicine by its unique clinical diagnosis and treatment theory and its emphasis on “Qi” and “blood” as the two basic elements of human physiology [17–19]. Qi is a universal concept representing the equivalent of the essence of life. Each organ has an overall Qi, such as the kidney Qi or liver Qi. A “Qi deficiency” leads to weakness and physical decline whereas “Qi stagnation” can cause swelling and pain. Blood represents all human body fluids. A “blood deficiency” can lead to dullness and dizziness whereas “blood stasis” tends to cause hemorrhage. In the pathology of pregnancy loss, the lack of kidney and liver function plays an important role. The kidney stores the necessary Qi for mobilizing and stimulating all other organs. It is responsible for regeneration and reproduction. The liver stores blood and regulates the flow of Qi and keeps breeding. In the theory of TCM, pregnancy loss can be caused by Qi deficiency, blood fever, blood deficiency, and “kidney deficiency”. In clinical practice, the most common cause of pregnancy loss is kidney deficiency, and women with this diagnosis often have a miscarriage early in pregnancy [20].

BSAT has a centuries-long history as a TCM. The concept of kidney-reinforcing was first proposed by Fu Qingzhu (1607–1684), a famous gynecologist from the Qing Dynasty. In his book “Fu Qingzhu Nv Ke”, it is mentioned that “once the kidneys are full and the uterus is more likely to ingest the essence of the innate, and once the blood is filled and the uterus is more likely to contain substances.” In the thirteenth chapter of “Nv Ke Bao Jian”, “Fetal leak, fetal restlessness,” it is mentioned that BSAT can be used for Qi and blood stasis type fetal movement restlessness (i.e., pregnancy loss), which has the effect of benefiting Qi and blood, and tonifying kidney and stabling fetus. BSAT has been used in our center for 5 years. Because its original compound contains an expensive medicinal ingredient,* Panax Ginseng C. A. Mey. *(Renshen in Chinese), we removed* Ginseng* and retained the remaining eight Chinese herbal ingredients in the original dosage ratio.

BSAT's mechanism of action is to nourish the kidneys to correct kidney deficiency and ultimately prevent pregnancy loss. However, the underlying mechanism of its therapeutic effect remains unclear. The reported mechanisms from modern researches of its various components are as follows:* Cuscutae Semen*,* Dipsaci Radix*, and* Herba Taxilli* are reported to have steroid-like and hormone-like effects, maintaining the hormone balance of the pregnant mother [21–23];* Paeoniae Radix Alba* and* Scutellariae Radix* have anti-inflammatory effects and improve immune balance [24, 25];* Cistanches Herba* and* Rhizoma Cibotii* maintain immune balance [26, 27];* Asini Corii Colla*, which has always been used as a tonic, promotes blood circulation [28]. In our study, we investigated the effects of BSAT on the maternal-fetal interface in the rat model of mifepristone-induced pregnancy loss.

## 2. Materials and Methods

### 2.1. Preparation of Modified BSAT Recipe

**Figure 1:**
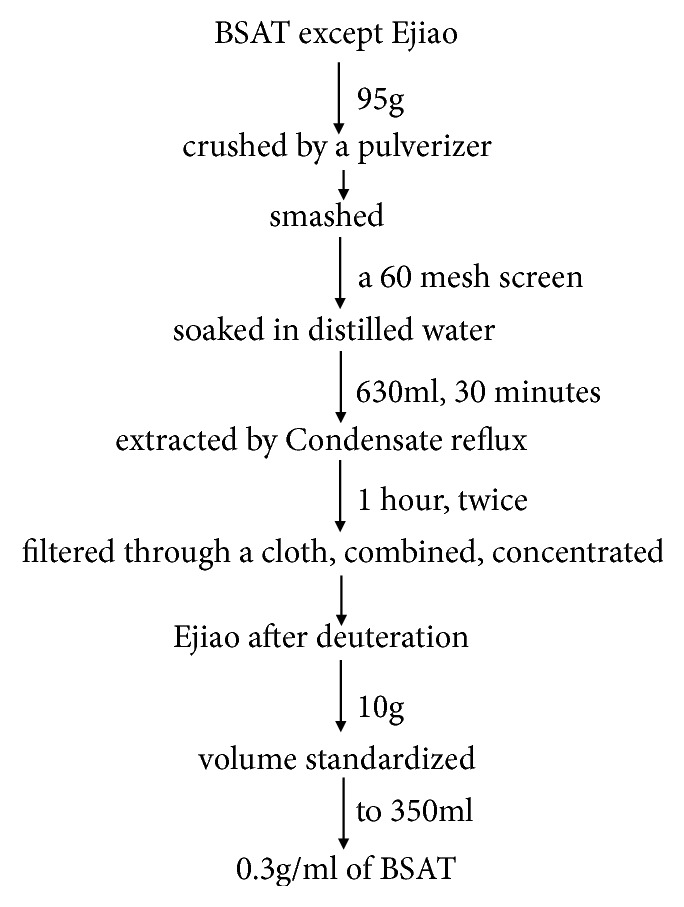
Extraction and purification protocol of BSAT.

The preparation procedure of BSAT is shown in Figure 1. The crude herbs (Table 1) of BSAT were purchased from Beijing Tong Ren Tang Chinese Medicine Co., Ltd. All the herbs except* Asini Corii Colla* were prepared in proportion by soaking in 630 ml pure water for 30 min and were extracted twice by refluxing for 1 h. The filtrate was merged, concentrated under vacuum and 10 g* Asini Corii Colla* was added after gelatinization. The volume was then adjusted to 350 ml with pure water to obtain a concentration of 0.3 g/ml BSAT.

### 2.2. HPLC Fingerprint Analysis

The HPLC fingerprinting of BSAT was constructed using Agilent 1200 liquid chromatograph and octadecylsilane bonded silica as the stationary phase (column: Waters Symmetry C 18, 4.6 250 mm, 5 mm). A gradient program was used consisting of 0.1% (V/V) phosphoric acid aqueous as mobile phase A and acetonitrile as mobile phase B. A linear gradient program was set as follows: 0–20 min, 80% A, 20–30 min, 70% A, 30–40 min, 60% A, 40–50 min, 50% A, 50-60 min, and 30% A. The flow rate was kept constant at 0.8 ml/min. The column temperature was maintained at 30°C and detection wavelength was set at 245 nm. The sample injection volume was 10 *μ*l.

### 2.3. Animal Treatment

Female Sprague Dawley rats, 8-10 weeks old, weighing 220 ± 20 g, were purchased from Beijing Huakang Biotechnology Co., Ltd. (license number: SCXK (Beijing) 2014-0004). All rats were housed in a specific pathogen-free (SPF) facility with a controlled temperature of 25°C and 12 h of circulating light (12 h light, 12 h dark). All animal treatment was performed in accordance with protocols approved by the Ethics Committee of Shengjing Hospital affiliated with China Medical University (2017PS284K).

Mifepristone tablets were purchased from Zhejiang Xianyi Pharmaceutical Co., Ltd. (H10950347): 25 mg/tablet, dissolved in 0.2 ml absolute ethanol, and added with 20 ml of 0.9% sodium chloride saline. Finally, a solution of 1.25 mg/ml was prepared for model establishment.

After one week of adaptive feeding, the estrus period was determined by vaginal smear between 8:00 and 9:00 p.m. daily. Males and females were caged at 2:1, and the vagina was examined between 8:00 and 9:00 a.m. the next day. Inseminated females were assigned to the 0.5th day of pregnancy (D 0.5). Rats with confirmed pregnancy were then randomly divided into three groups: a BSAT group, a model group, and a control group. The BSAT group was administered 6.3 ml/kg BSAT (i.e., 1.89 g/kg, calculated by adults weighing 60 kg) at D 0.5-D 10.5 8:00–9:00 a.m. and 8:00–9:00 p.m. continuously, and 1.25 mg/kg mifepristone was administered at D 10.5 12:00 a.m. The model group replaced BSAT with an equal volume of normal saline, and the amount of mifepristone was the same as the BSAT group. The control group replaced both BSAT and mifepristone with an equal volume of normal saline. At D 13.5, the rats were anesthetized with an excess dose of pentobarbital. The placenta was taken and hemisected using a double-edged razor blade, and then each half was immediately fixed in 4% paraformaldehyde and stored at 4°C. The decidua tissue was also taken and stored in a dry tube and stored at -80°C.

### 2.4. Number of Surviving Embryos and Embryo Resorption Rate

We evaluated the number of surviving embryos and the rate of embryo absorption by observing the morphology of the uterus. Embryos were characterized as either a surviving embryo: no blood was present in the uterus, the embryo was well developed, and the individual was large and reddish; or an absorbed embryo: the uterus was “bamboo-like”, the embryo volume was significantly reduced, the embryo in the uterus was dark brown, and the fetal placenta exhibited obvious bleeding or necrosis. Embryo resorption rate = number of absorbed embryos/(number of absorbed embryos + number of surviving embryos) × 100%.

### 2.5. Hematoxylin-Eosin Staining Analysis

To analyze placental morphology, tissues were collected from the three groups on D 13.5 and fixed in 4% paraformaldehyde for 12-16 h. After the tissue samples were fixed, they were dehydrated, embedded in paraffin, and serially sectioned at a thickness of 5 *μ*m. The first cut section was taken as the largest cross-section of the placental tissue and mounted on a glass microscope slide. Conventional hematoxylin-eosin (HE) staining was performed for histological examination, which was analyzed under an optical microscope. Image pro plus (IPP) software (Media Cybernetics, Kunshan, USA) was used to calculate the total area of the largest cross-section of the placenta and the area ratio of each placenta zone, decidua basalis (db), the junctional zone (Jz), and the labyrinth zone (Lz), respectively.

### 2.6. Immunohistochemical Analysis

For immunohistochemistry (IHC) analysis, 3.5 *μ*m sections were cut, deparaffinized, rehydrated, and then incubated in 3% H_2_O_2_ for 30 min at 37°C. Then the sections were blocked using goat serum (1:20, ZSGB-BIO, China) for 30 min at 37°C. The sections were labeled with the following primary antibodies: Ki67 antibody (1:100, abcam#ab16667, Cambridge, UK), ER*α* antibody (1:100, abcam#ab32063, Cambridge, UK) and PR antibody (1:100, abcam#ab16661, Cambridge, UK) for 12 h at 4°C. Then, the sections were incubated with tagged goat-anti-rabbit secondary antibody for 2 h at room temperature. The DAB (1:20, ZSGB-BIO, China) reaction results were imaged using a Nikon ECLIPSE 80i (Nikon, Japan).

### 2.7. Western Blot Analysis

The decidua was treated with 300 *μ*l of radioimmunoprecipitation assay (RIPA) buffer combined with protease inhibitor (PMSF) (Beyotime #P0013B, #ST506, China). The lysates were collected by centrifugation at 14,000 rpm for 20 min at 4°C. Total protein was qualified using a bicinchoninic acid assay kit (Beyotime, #P0010S, China), with samples diluted to 4 *μ*g/*μ*l. Protein samples were separated by 8% sodium dodecyl sulfate-polyacrylamide gel electrophoresis (Beyotime, China); 0.45 *μ*m polyvinylidene difluoride membranes (EMD Millipore, USA) were used for transfer; and 5% nonfat powdered milk dissolved in TBST (A buffer solvent containing Tris-Hcl, NaCl and tween20) was used for blocking. Thereafter, all membranes were incubated for 12 h at 4°C with the following primer antibodies: ER*α*, 1:500, absin#123979, China; PR, 1:500, absin#119762, China; Akt, 1:1000, CST#4691, MA, USA; Phosopho-Akt, 1:1000, CST#13038, MA, USA; Erk1/2 1:1000, CST#4695, MA, USA; Phospho-Erk1/2 1:1000, CST#4370, MA, USA. Subsequently, the membranes were incubated on the following day with secondary antibody (ZSGB-BIO, China) for 1 h at 25°C. Thereafter, the blots were added to BeyoECL Star (Thermo Fisher Scientific, #1862420, #1862421, USA) blotting substrate and visualized by C300. The gray densitometric was analyzed using Image J (National Institutes of Health, USA).

### 2.8. Quantitative Reverse Transcription Polymerase Chain Reaction Analysis

Decidua tissues were used to extract total RNA with RNAiso Plus (Takara #9108, Japan), and the expression of ER*α* and PR was analyzed. The RNA was reverse transcribed into cDNA using a PrimeScript RT Regent Kit (Takara #RR047A, Japan) according to the manufacturer's protocol following by the RT reaction that as 37°C for 15 min, 85°C for 5 s and 4°C. All the cDNA was stored at −20°C. Quantitative PCR was performed using SYBR Premix Ex Taq ii (Takara #RR820A, Japan). The primers used in this study are listed in Table 2. Quantitative RT-PCR was conducted at 95°C for 30 s followed by 40 cycles at 95°C for 5 s and 60°C for 34 s and final extension at 60°C for 15 s in 7500 software v 2.0.6 (Life Technologies, USA). The relative levels of mRNA were normalized with Gapdh; gene expression was analyzed by 2^-ΔΔCt^.

### 2.9. Statistical Analysis

All results were analyzed with GraphPad Prism 7.0 using the two-tailed unpaired t test. Count data were expressed as the mean ± SEM. P < 0.05 was considered a statistically significant difference.

## 3. Results

### 3.1. Preparation of BSAT and Analysis of Its Quality Attributes

**Figure 2:**
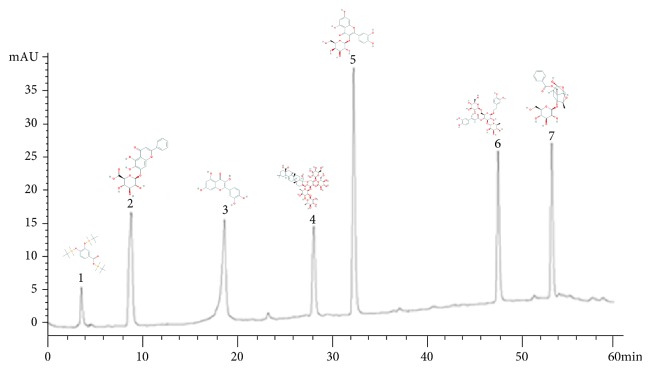
HPLC analysis of BSAT. Numbered peaks were echinacoside, baicalin, quercetin, saponin B, hyperoside, protocatechuic acid, and paeoniflorin, respectively.

BSAT was prepared according to an in-house optimized extraction and purification protocol (see Materials and Methods, Figure 1). HPLC analysis determined the content of the signature compounds of each BSAT ingredient except for the main compound,* Asini Corii Colla*, L-hydroxyproline (Figure 2). As shown in Table 1, the content of saponin B and quercetin was above 1.2 mg/g, whereas the content of hyperoside, paeoniflorin, and baicalin was up to 0.5 mg/g. The HPLC analysis indicated that the optimized extraction method did not eliminate the main compounds of BSAT.

### 3.2. BSAT Reduced Embryo Resorption Rate in the Rat Model of Pregnancy Loss

**Figure 3:**
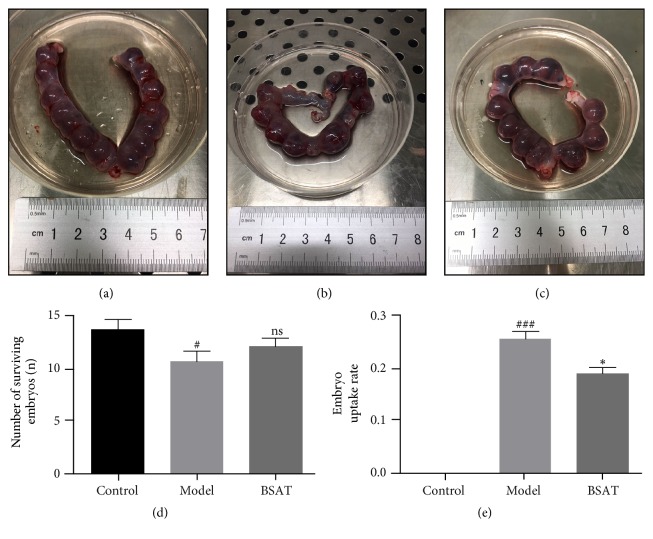
Number of surviving embryos and embryo resorption rate. The number of surviving embryos and embryo resorption rate were calculated on D 13.5. (a) Uterus anatomy image of control group. (b) Uterus anatomy image of model group. (c) Uterus anatomy image of BSAT group. (d) Number of surviving embryos of each group. (e) Embryo resorption rate of each group. The data were shown as the mean ± SEM (n = 8). ns P≥0.05 compared with model group, *∗∗* P<0.01 compared with model group, # P<0.05 compared with control group, and ### P<0.001 compared with control group.

We used the mifepristone-induced pregnancy loss model in pregnant rats to investigate the effect of BSAT on embryo survival and resorption rates. Interestingly, although BSAT administration to the mifepristone-treated pregnant rats did not significantly increase the embryo survival rate (Figure 3(d), P = 0.3097), it did significantly reduce the embryo resorption rate (Figure 3(e), P = 0.0024).

### 3.3. BSAT Increased the Maximum Cross-Sectional Area and the Labyrinth Zone (Lz) of the Placenta in the Rat Model of Pregnancy Loss

**Figure 4:**
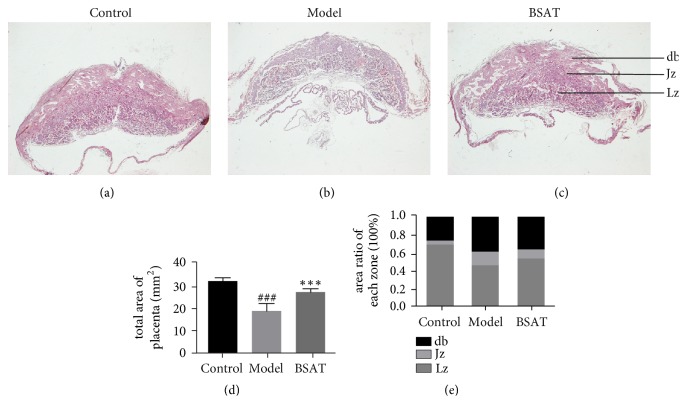
HE staining of placenta. (a-c) Representative placental H&E staining for each group. (d) The maximum cross-sectional area of placenta. (e) The area ratio of Lz. The data were shown as the mean ± SEM (n = 8). *∗∗∗* P<0.001 compared with model group; ^###^ P<0.001 compared with control group. Decidua basalis (db), junctional zone (Jz), and labyrinth zone (Lz).

To assess the development of the placenta in the pregnancy loss model, a histological analysis was performed on placenta tissue samples using HE staining (Figure 4). We found that the maximum cross-sectional area of the placenta on D 13.5 was significantly reduced in the model group (Figure 4(d), P<0.001) as compared to that in the control group, while the BSAT group was significantly higher than that in the model group (Figure 4(d), P<0.001). A similar trend was observed in the three groups for the area ratio of Lz (Figure 4(e)). Our observations indicated that mifepristone caused pregnancy loss phenotype by blocking the placental development at the maternal-fetal interface, whereas BSAT appeared to improve stagnation or stunted growth.

### 3.4. BSAT Improved the Expression of ER*α* and PR in the Lz in the Pregnancy Loss Rat Model

**Figure 5:**
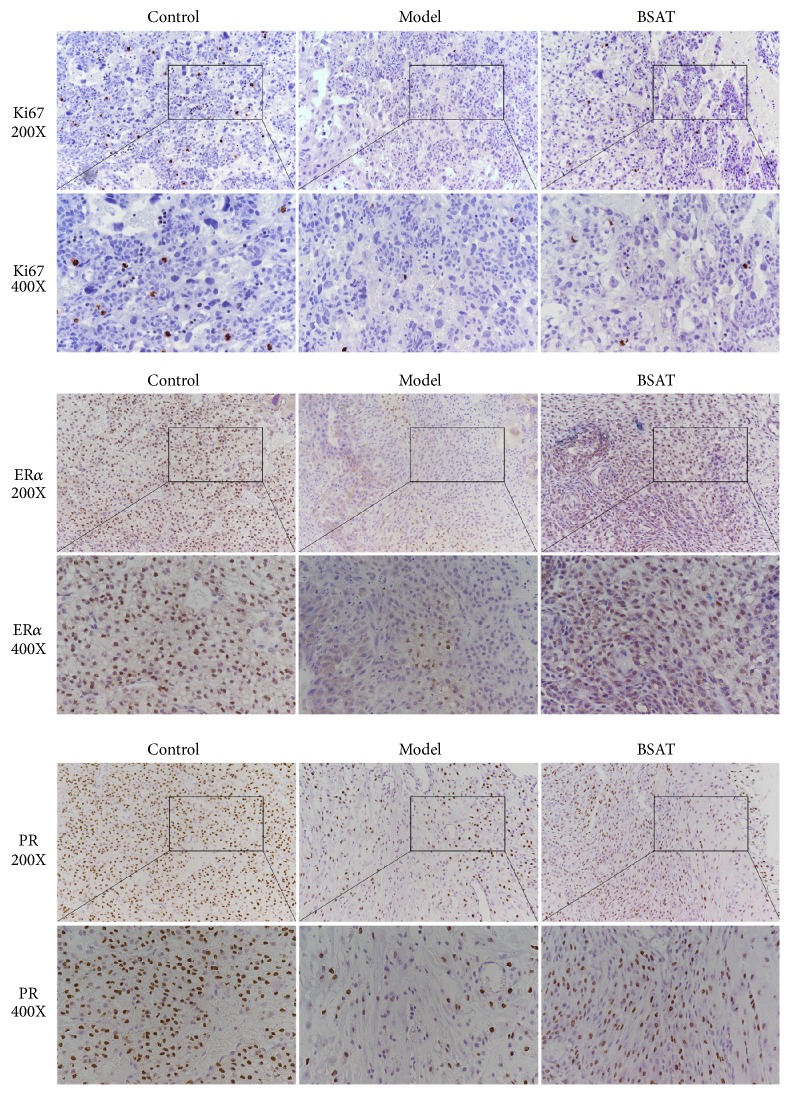
IHC staining of the Lz in placenta. The upper two rows were the expression of Ki67 in the labyrinth, which are 200-fold and 400-fold, respectively. The middle two rows were the expression of ER*α* in the labyrinth, which are 200-fold and 400-fold, respectively. The bottom two rows were the expression of PR in the labyrinth, which are 200-fold and 400-fold, respectively. The brown colored nuclei indicate the expression of the target protein, and the blue nuclei suggest that the target protein is not expressed.

Next, we used IHC staining to examine the expression of nuclear ER*α* and PR in the Lz. As shown in Figure 5, the amount of ER*α* and PR positive cells in the Lz was down-regulated by mifepristone administration. However, the amount of Ki67, ER*α* and PR positive cells in the Lz were significantly higher in the BSAT group than those in the model group. The results confirmed the role of mifepristone as a progesterone receptor antagonist and indicated the potential effect of BSAT as a potential stimulator for estrogen and progesterone receptor expression.

### 3.5. BSAT Increased the Protein Expression of ER*α*, PR, Phospho-Akt/Akt, and Phospho-Erk1/2/Erk1/2 in the Decidua of the Pregnancy Loss Rat Model

**Figure 6:**
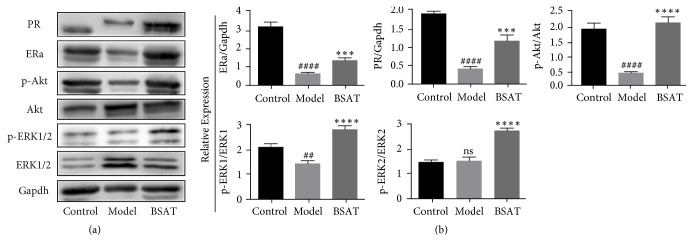
Western blot analysis in decidua tissue. (a) BSAT improved phosphorylation of Akt and Erk1/2 pathways and enhanced ER*α* and PR expression. (b) Gray level analysis of Western blots. Data was shown as mean ± SEM, *∗∗∗* P<0.001 compared with model group, *∗∗∗∗* P<0.0001 compared with model group, ## P<0.01 compared with control group, and #### P<0.0001 compared with control group.

After determining the presence of ER*α* and PR in the maternal-fetal interface of the placenta, we investigated the protein expression levels of ER*α* and PR as well as the classical Akt and Erk1/2 pathways in the decidua (Figure 6(a)). Mifepristone-induced pregnancy loss was associated with reduced protein levels of ER*α*, PR, p-Akt/Akt, and p-Erk1/2/Erk1/2 in the decidua of the model group rats (Figure 6(b), P<0.0001, P<0.0001, P = 0.0012, P = 0.7950, and P<0.0001, respectively). Although these levels were partially restored in the decidua of the BSAT group, they failed to reach the protein levels recorded in that of the control group (Figure 6(b), P = 0.0001, P = 0.0005, P<0.0001, P<0.0001 and P<0.0001, respectively).

### 3.6. BSAT Improved the mRNA Expression of ER*α* and PR in the Decidua in the Pregnancy Loss Rat Model

**Figure 7:**
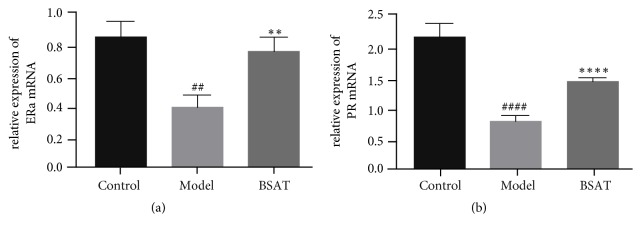
Quantitative RT-PCR analysis in decidua tissue. (a) Relative expression of ER*α* mRNA. (b) Relative expression of PR mRNA. Data was shown as mean ± SEM, *∗∗* P<0.01 compared with model group, *∗∗∗∗* P<0.0001 compared with model group, ## P<0.01 compared with control group, and #### P<0.0001 compared with control group.

Next, we analyzed the expression of ER*α* and PR mRNA levels in decidua samples by quantitative RT-PCR analysis (Figure 7). Consistent with the results of the protein level analysis, the mRNA levels of ER*α* and PR decreased significantly in the model group (P = 0.0078, P<0.001, respectively), but increased in the BSAT group (P = 0.0032, P = 0.001, respectively).

## 4. Discussion

In our study, we used pregnant rats exhibiting a mifepristone-induced pregnancy loss model to demonstrate that BSAT treatment reduces the pregnancy loss effects caused by mifepristone administration.

Chinese medicine practitioners prescribe TCM according to the principle of “Jun, Chen, Zuo, and Shi” which means emperor, minister, assistant, and messenger, respectively. Each herb or other type of ingredient has its unique medicinal properties and potential interactions with other ingredients. The guiding principle for creating a TCM is to reduce or avoid the side effects and toxicity of other herbal ingredients, enhance its medicinal properties, and, thus, achieve a holistic and harmonious equilibrium with a direct and accurate therapeutic effect on a disease.

In our experiment, BSAT was prepared from eight different medicinal materials. HPLC analysis detected seven out of eight ingredient-specific signature compounds. Hypericin is derived from the mature seeds of* Cuscutae Semen* (the role of Jun, or emperor, in BSAT) and quercetin is derived from the dry stems of the* Herba Taxilli* (also the role of Jun). Saponin B, paeoniflorin, baicalin, and protocatechuic acid are derived from the dry roots of* Dipsaci Radix*,* Paeoniae Radix Alba*,* Scutellariae Radix* and* Rhizoma Cibotii*, respectively, which all have the role of Chen, or minister, in BSAT. L-hydroxyproline is derived from the* Asini Corii Colla* (the role of Zuo, or assistant), while echinacoside is derived from the dried succulent stems of* Cistanches Herba* (the role of Shi, or messenger).

Mifepristone, one of the earliest approved drugs for medical abortion, is widely used to terminate an early or mid-term pregnancy [29]. In addition, it has been successfully used for the treatment of certain mental illnesses in recent years [30]. In animal experiments, it is also used as the inducing agent in pregnancy loss models due to its activity as a progesterone and glucocorticoid receptor antagonist [31]. Studies investigating the molecular interaction involving mifepristone have shown that it perturbs the immune functions at the maternal-fetal interface, including the immune responses of lymphocytes and natural killer cells [32]. However, mifepristone treatment regimens tested in various animal experiments vary widely, as does the embryo resorption rate [33–35].

We tested the effect of different doses of mifepristone on the embryo resorption rate in pregnant rats and found that 5 mg/kg of mifepristone caused 100% embryo resorption, which is suitable for establishing a complete pregnancy loss model; at 3.75 mg/kg and 2.5 mg/kg of mifepristone, the embryo resorption rate was 82-90% and 48-57%, respectively; at 1.25 mg/kg of mifepristone, however, a much lower embryo resorption rate of 16-25% was obtained (Table 3). In order to establish a robust model of pregnancy loss in clinical practice, the dose of 1.25 mg/kg of mifepristone was used in our subsequent experiments.

In our experiment, the transgastric administration of BSAT significantly decreased the embryo resorption rate, although the number of surviving embryos did not change significantly. This suggested that BSAT seemed to improve the mifepristone-induced imbalance at the maternal-fetal interface. Mifepristone functions as a progesterone receptor antagonist. However, due to the flavonoid content of BSAT, its continued administration might stimulate the progesterone receptors at the maternal-fetal interface, which would create a protection against the antagonistic effect of mifepristone. This was the most pronounced phenotypic change indicated by the protective effect of BSAT against pregnancy loss.

It is well known that the growth of the fetus and the maintenance of the pregnancy depends on the nutritional supply via the placenta. Factors such as placental size, morphology, and blood flow play important roles in placental nutrition transfer [36]. As an animal model for studying the maternal-fetal interface, the rat placenta consists of three zones with different shapes and functions: the db, the Jz, and the Lz. The db as the major site for hormone secretion consists of trophoblasts and maternal blood vessels, but no fetal blood vessels. The presence of Jz is essential for embryo viability, but no clear physiological function has been assigned to it. The Lz, as the layer most sensitive to external disturbances, consists of trophoblasts and both maternal and fetal blood vessels and, therefore, functions as the main site of maternal-fetal exchange [5, 37].

In our study, the Lz exhibited a dynamic change in proportion during the entire gestation period [38]. On D 14.5, it started to show a significant difference as compared with the other zones, and reached a maximum on D 16.5. Its volume expansion was associated with an increase in maternal vascular permeability and fetal capillary volume.

In earlier studies, the placental weight was often used as an indicator for evaluating placental development [39]. However, because the placenta has a loose vascular plexus structure, its wet or dry weight can be affected during experimental processing steps or from humidity. In our experiment, the maximum cross-sectional area of the placenta after tissue fixation was chosen as the evaluation index because the placenta is a hemispherical structure, and, therefore, select dimensions of the fixed, cross-sectional area can be used as robust indicators for evaluating placenta development.

Estrogen and progesterone are indispensable factors of the female reproductive system that control physiological functions during the menstrual cycle, embryo implantation, pregnancy, and childbirth [40]. Estrogen functions in target tissues via binding to ER*α*, which activates ER*α* by the dimerization required for its translocation to the nucleus where it acts as a DNA-binding transcription factor. During early embryo implantation, ER*α* controls decidualization by paracrine action. Paracrine effects of estrogen receptors can stimulate secretion of fibroblast growth factors (FGFs) and insulin-like growth factor-1 (IGF1). The FGF family can activate Erk1/2 signaling cascades in target cells, thereby stimulating cell proliferation [41]. IGF1 activates the PI3/Akt pathway, regulates phosphorylation and inactivation of glycogen synthase kinase 3 beta (GSK3*β*), and maintains target cell proliferation [42]. PR, which specifically binds progesterone, is present in the female reproductive system where it is involved in cell proliferation and differentiation. Estrogen-activated ER*α* directly regulates the expression and activity of PR [43, 44]. In addition, the binding of progesterone to the PR activates the cytoplasmic MAPK/Erk signaling pathway [44, 45], resulting in a high expression of Erk1/2 and decidualization in human and rodent uteri [46–48]. The regulation by steroid hormones is indirectly reflected in the changes in hormone receptor target genes [49]. Previously, it has been reported that patients with endometriosis, endometrial hyperplasia, or endometrial cancer have a certain level of progesterone resistance and a reduced expression of PR in uterine [6, 50]. Therefore, in addition to analyzing protein expression by Western blot and protein localization using antibodies in IHC, we also analyzed the mRNA expression of ER*α* and PR in the decidua by RT-PCR. Our experiments confirmed the increase in ER*α*, PR, and p-Akt/Akt, p-Erk1/2/ Erk1/2 expression in the BSAT group, elucidating the effect of BSAT on embryo resorption rate and placental development.

## 5. Conclusions

**Figure 8:**
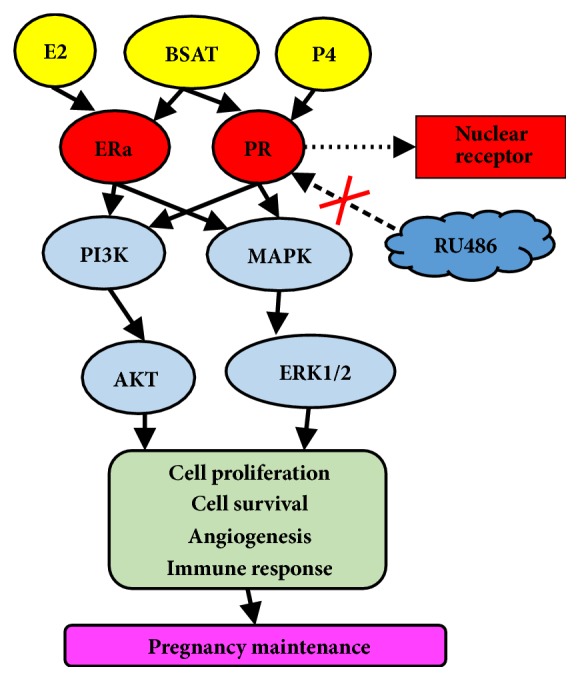
The possible mechanism of action of BSAT on mifepristone-induced pregnancy loss rat model at the maternal-fetal interface. Estrogen (E2), modified Bushen Antai recipe (BSAT), progesterone (P4), estrogen receptor alpha (ER*α*), progesterone receptor (PR), and mifepristone (RU486).

In summary, our results indicated that BSAT acted on pregnancy loss in a rat model via an increase in expression of ER*α* and PR in the decidua of the maternal-fetal interface. The upregulation of PR might affect the MAPK/Erk pathway, which is involved in inflammatory response and cytokine production. Simultaneously, the upregulation of ER*α* might affect the PI3K/Akt and MAPK/Erk signaling cascade and contributes to cell expansion and survival. Hence, BSAT promoted the maintenance of placental development and exerted a protective effect on pregnancy loss (Figure 8).

## Figures and Tables

**Table 1:** Composition of BSAT.

	Latin name	Chinese pinyin name	Main ingredient	PubChem CID	Content in BSAT (mg/g)
1	*Cuscutae Semen*	Tu-Si-Zi	Hyperoside	5281643	0.54752407
2	*Dipsaci Radix*	Chun-Xu-Duan	Saponin B	3081325	1.224066956
3	*Herba Taxilli*	Sang-Ji-Sheng	Quercetin	5280343	1.279052221
4	*Paeoniae Radix Alba*	Bai-Shao	Paeoniflorin	442534	0.45909865
5	*Scutellariae Radix*	Huang-Qin	Baicalin	64982	0.868340481
6	*Cistanches Herba*	Rou-Cong-Rong	Echinacoside	5281771	0.030497498
7	*Rhizoma Cibotii*	Gou-Ji	Protocatechuic acid	528594	0.018787507
8	*Asini Corii Colla*	E-Jiao	L-hydroxyproline	69248	undetected

**Table 2:** Primer sequences for RT-PCR analysis.

Gene	Forward Premier	Reverse Premier
ER*α*	ATGACCTGCTGCTGGAGATG	GTGCTGAAGTGGAGCTGGTG
PR	GCCACTCATCAACCTGCTCA	CTGCCTCTCGCCTAGTTGGT
Gapdh	TGGTGAAGGTCGGTGTGAAC	GACTGTGCCGTTGAACTTGC

**Table 3:** The effect of gradient dose of mifepristone on the embryo absorption rate of pregnant rats.

Dose of RU486	5 mg/kg	3.75 mg/kg	2.5 mg/kg	1.25 mg/kg
Number of rats	6	6	6	6
Surviving embryo number	0 ± 0	1.8 ± 0.581	5.6 ± 0.5099	10.8 ± 0.8602
Uptake embryo number	10.2 ± 1.356	10.8 ± 1.114	6.4 ± 0.9274	2.8 ± 0.5831
Embryo uptake rate (%)	100 ± 0	86.06 ± 4.803	52.49 ± 4.776	20.79 ± 4.276

Data was shown as mean ± SEM.

## Data Availability

The figures and tables data used to support the findings of this study are included within the article. The original data used to support the findings of this study are available from the corresponding author upon request.

## Abbreviations

BSAT: Modified Bushen Antai recipe
HPLC: High-performance liquid chromatography
HE: Hematoxylin and eosin staining
IHC: Immunohistochemical staining
ER*α*: Estrogen receptor alpha
PR: Progesterone receptor
HCG: Human chorionic gonadotropin
TCM: Traditional Chinese medicine
db: Decidua basalis
Jz: Junctional zone
Lz: Labyrinth zone.
